# Endotoxin and Cancer

**DOI:** 10.1289/ehp.0800439

**Published:** 2009-05-07

**Authors:** Jessica I. Lundin, Harvey Checkoway

**Affiliations:** Department of Environmental and Occupational Health Sciences, University of Washington School of Public Health, Seattle, Washington, USA

**Keywords:** cancer, carcinogenesis, endotoxin, epidemiology, lipopolysaccharide, LPS, lung cancer, occupational epidemiology

## Abstract

**Objective:**

Exposure to endotoxin, a component of gram-negative bacterial cell walls, is widespread in many industrial settings and in the ambient environment. Heavy-exposure environments include livestock farms, cotton textile facilities, and saw mills. Concentrations are highly variable in non-occupational indoor and outdoor environments. Endotoxin is a potent inflammagen with recognized health effects, including fever, shaking chills, septic shock, toxic pneumonitis, and respiratory symptoms. Somewhat paradoxically, given the putative role of inflammation in carcinogenesis, various lines of evidence suggest that endotoxin may prevent cancer initiation or limit tumor growth. The hypothesis that components of bacteria may retard cancer progression dates back to William B. Coley’s therapeutic experiments (“bacterial vaccine”) in the 1890s.

**Data sources:**

In this article, we review epidemiologic, clinical trial, and experimental studies pertinent to the hypothesis that endotoxin prevents cancer. Since the 1970s, epidemiologic studies of cotton textile and other endotoxin-exposed occupational groups have consistently demonstrated reduced lung cancer risks. Experimental animal toxicology research and some limited therapeutic trials in cancer patients offer additional support for an anticarcinogenic potential. The underlying biological mechanisms of anticarcinogenesis are not entirely understood but are thought to involve the recruitment and activation of immune cells and proinflammatory mediators (e.g., tumor necrosis factor α and interleukin-1 and -6).

**Conclusions:**

In view of the current state of knowledge, it would be premature to recommend endotoxin as a cancer-chemopreventive agent. Nonetheless, further epidemiologic and experimental investigations that can clarify further dose–effect and exposure–timing relations could have substantial public health and basic biomedical benefits.

Endotoxins are integral components of the outer membrane of gram-negative bacteria cell walls, composed of proteins, lipids, and lipopolysaccharide (LPS), which are released when bacteria lyse (Campbell et al. 2008). LPS is considered to be responsible for most of the biological properties of bacterial endotoxins, particularly the lipid component (lipid A, a phosphoglycolipid) (Hodgson 2006; Reisser et al. 2002). Endotoxins are a contaminant of various organic dusts and other environmental media that support gram-negative bacterial growth [Centers for Disease Control and Prevention (CDC) 2006; Gehring et al. 2004; Liebers et al. 2006; Park et al. 2006]. The bacterial constituents are continuously shed into our surrounding environment; consequently, exposure to endotoxin is extremely widespread.

The *Limulus* amoebocyte lysate (LAL) assay for environmental endotoxin levels was adopted as the standard assay of endotoxin detection by the U.S. Food and Drug Administration in the 1980s (Liebers et al. 2006). This assay is based on the activation of a clotting enzyme in the lysate. Endotoxin levels are often expressed as endotoxin units (EU; 1 EU ≈ 0.1 ng, depending on the reference standard), or as concentration of endotoxin per milligram of dust or per cubic meter of air. Of note, LAL tests are not internationally standardized, and measurements may vary among laboratories (Liebers et al. 2006).

Of particular interest from a health effects perspective are the more intense exposures experienced in numerous manufacturing and agricultural settings throughout the world. Substantial endotoxin exposure occurs in agricultural work, garbage handling, sewage treatment, and incineration industries, textile industries (particularly cotton products factories), and saw mills, and to a lesser degree in occupations with exposures to certain types of water-based metalworking fluids and in cigarette factories, fiberglass production facilities, and paper mills, among others (Astrakianakis et al. 2007; CDC 1998; Kuzmickiene et al. 2004; Liebers et al. 2006; Mandryk et al. 1999; Nieuwenhuijsen et al. 1999; Rapiti et al. 1997). Cotton factories in the Shanghai textile industry have been documented to have high endotoxin exposure concentrations (Astrakianakis et al. 2007). By way of illustration, the mean of the endotoxin levels that have been measured in representative cotton factories was 366 EU/m^3^ (range, 44–1,871 EU/m^3^) (Astrakianakis et al. 2006a). Additionally, reported mean endotoxin concentrations of 40 and 48 EU/m^3^ have been reported among municipal waste management workers (Spaan et al. 2006; Wouters et al. 2006). In the agricultural industry, an overall mean endotoxin concentration of 230 EU/m^3^ has been reported, with mean measurements of 2,700 EU/m^3^ (range, 96–42,300 EU/m^3^) in the grain, seeds, and legume primary production sector and 1,190 EU/m^3^ (range, 62–8,120 EU/m^3^) in the primary animal production sector (Spaan et al. 2006). Other studies have reported endotoxin levels for livestock farmers ranging from 11 to 159 EU/m^3^ and field crop and fruit farming exposure levels ranging from low to > 1,500 EU/m^3^ (Nieuwenhuijsen et al. 1999), and an exposure concentration of 140 EU/m^3^ among swine farmers (Chang et al. 2001).

Endotoxin is ubiquitous in the environment, although the exposure in occupational settings, frequently > 100 ng/m^3^, is more intense than exposure in the home, < 1 ng/m^3^ (Rylander et al. 1989). Nonetheless, adverse health effects have been observed at endotoxin levels as low as 0.2 ng/m^3^ (Smid et al. 1992). The human health effects of acute exposure to endotoxin include sepsis; clinical symptoms such as fever, shaking chills, and septic shock; and, at lower doses, toxic pneumonitis, lung function decrements, and respiratory symptoms, such as byssinosis (“Monday morning chest tightness”) (Rylander 2002, 2006). Chronic exposures have been related to the risk of developing nonatopic chronic obstructive pulmonary diseases (Schwartz et al. 1995; Smid et al. 1992; Wang et al. 2005) and to the severity of asthma (Michel et al. 1996). In contrast, numerous studies have demonstrated seemingly protective effects of environmental endotoxin exposure on atopic asthma risk and allergy development in early childhood (Remes et al. 2003; von Mutius et al. 2000), and atopy in adults (Eduard et al. 2004; Gehring et al. 2004; Portengen et al. 2005). As we discuss in some detail in this article, an inverse association with endotoxin exposure and the risk of cancer of the lung, and potentially other cancer end points, has consistently been demonstrated.

More than a century of clinical, laboratory, and epidemiologic research demonstrates that endotoxin has antitumor properties (Liebers et al. 2008; Liu 2002), but an understanding of the underlying mechanisms, and the subsequent development of an effective therapeutic application of endotoxin, has yet to be elucidated. We reviewed current and historical literature identified in Medline (National Library of Medicine 2006) electronic database, 1973–2008, using combinations of search key words such as endotoxin, LPS, epidemiology, lung, cancer, farmer, textile, and cotton. The text and citations of all identified supporting articles were reviewed with a particular focus on lung cancer, cotton textile workers [studies of textile workers that did not specify type of textile (i.e., cotton) were not reviewed], and studies of farmers by type of farming (dairy, crop, etc.). In addition a Medline search of publications from 1990 to 2008 was performed that reviewed the underlying mechanism of action so as to best describe the paradoxical understanding and association of the immune system response to endotoxin exposure and cancer.

In this review we discuss the historical and current understanding of the association of endotoxin exposure and cancer, therapeutic uses/treatment of cancer with LPS, epidemiologic studies of endotoxin exposure, and the underlying mechanisms to explain the human studies.

## Endotoxin and Cancer

### Early experiments

In the late 19th century, William B. Coley, with the assistance of established anecdotal theories of the beneficial effect of fever on tumors (McCarthy 2006), recognized regression and, in some cases, necrosis of tumors in advanced cancer patients suffering concomitant bacterial infections. Coley went on to successfully treat cancer in terminally ill patients by injecting mixed bacterial toxins in and around the tumors (Coley 1894). Despite the successes, this treatment was discontinued because the anticancer effect in patients was not consistent and repeated injections caused severe side effects, such as high fever and chills, that were not yet understood (Mueller 1998). In the early 1940s, LPS was identified as the active ingredient in Coley’s “bacterial vaccine,” and the antitumor effects of the bacterial polysaccharide were successfully demonstrated *in vivo* (Shear and Perrault 1944; Shear and Turner 1943). When isolated LPS was found to be ineffective as an antitumor agent in culture, it was determined that the effects were mediated by host-dependent mechanisms. Almost three decades later, tumor necrosis factor α (TNF-α) was determined to be the effective agent with antitumor properties (Carswell et al. 1975). By the mid-1980s therapeutic uses of TNF-α were being tested, but the therapy was less effective than hoped and caused undesired side effects, such as headache, nausea, vomiting, fever, hypotension, and diarrhea (Clark 2007; Mueller 1998; Spriggs et al. 1988). Around this same time, it was discovered that TNF-α was identical to cachectin, a mediator responsible for cachexia associated with sepsis (Clark 2007; Ghezzi and Cerami 2005). The adverse effects of TNF-α were quickly accepted as limitations to its direct use as an antitumor agent (Ghezzi and Cerami 2005; Mueller 1998).

### Treatment of cancer with LPS

Laboratory studies have successfully demonstrated therapeutic effects when administering LPS, or synthetic lipid A molecule, including inhibition of tumor size and growth (Andreani et al. 2007; Chicoine et al. 2001; Kuramitsu et al. 1997; Morita et al. 1996). Morita et al. (1996) demonstrated this effect to be dose dependent. Additionally, an increased survival time has been noted for mice infected with cancer cells that have been inoculated with LPS (Andreani et al. 2007; Lange 1992). An inverse dose–response association was demonstrated on the survival of cancer-bearing rats that were administered a synthetic analogue of lipid A (Kuramitsu et al. 1997). Furthermore, antigenic memory has been demonstrated on mice with tumor cells planted intracranially; the mice with previous LPS-eradicated tumors showed increased survival compared with those without previous tumors (Won et al. 2003).

Subsequently small clinical trials administering LPS, or a lipid A analog, have been performed. Cancer remission and disease stabilization have been demonstrated in cancer patients (de Bono et al. 2000; Engelhardt et al. 1991; Goto et al. 1996; Otto et al. 1996). However, clinical toxicities have been unavoidable, even with the pretreatment of ibuprofen (de Bono et al. 2000; Engelhardt et al. 1991; Otto et al. 1996).

### Epidemiologic studies of endotoxin exposure and cancer risk

#### Lung cancer

Cancer risks, particularly lung cancer, have been investigated in relation to occupational endotoxin exposures (Table 1). Cotton textile and farming industries have been a particular focus of epidemiologic research because of the substantial endotoxin exposure in these occupational settings, so we review these two industries in detail. Findings from early occupational cohort studies demonstrated reduced risks for lung cancer among cotton textile workers in the United States (Henderson and Enterline 1973; Merchant and Ortmeyer 1981) and the United Kingdom (Hodgson and Jones 1990), particularly in those with longer durations of employment. These results were regarded as somewhat surprising when first observed. Lower than expected lung cancer risks were subsequently reported from a cohort study conducted among women textile workers in Shanghai (Astrakianakis et al. 2007; Wernli et al. 2003), a separate, unrelated, case–control study of both men and women in the cotton textile industry in Shanghai (Levin et al. 1987), cotton textile workers in Poland (Szeszenia-Dabrowska et al. 1999), and a study of Italian cotton mill workers (Mastrangelo et al. 2008). Slightly elevated lung cancer risks were noted in Lithuanian and Finnish cohorts of cotton textile workers (Koskela et al. 1990; Kuzmickiene et al. 2004); however, extended follow-up of the Lithuanian cohort, by 5 years, indicated significantly reduced lung cancer risk among male workers employed for at least 10 years (Kuzmickiene and Stukonis 2007), and the reported risk in the Finnish cohort was based on three cases. In a meta-analysis of studies of cotton workers published during or before 1990, and of studies published during or before 2002, lung cancer risk was significantly reduced (Mastrangelo et al. 2002). Of note, the risk estimate for lung cancer was closer to unity when the more recent studies were included. The authors of the meta-analysis hypothesized this may be due to a lowering of dust concentration in the workplace in recent years.

Protection for lung cancer has been demonstrated to be similar among different types of farming (Blair et al. 2005; Lee et al. 2002), although most studies reviewed demonstrated a greater protective effect in livestock farmers, specifically dairy farmers, compared with orchard/crop farmers (Laakkonen and Pukkala 2008; Lange et al. 2003; Mastrangelo et al. 1996, 2004; Pukkala and Notkola 1997; Reif et al. 1989); Lange et al. (2003) demonstrated that the risk difference was statistically significant. Additionally, crop farmer exposures are predominantly during warmer harvest months (~ 4 months) and may not be representative of the actual annual dose, whereas the exposure experience of livestock farmers occurs 12 months a year (Lange et al. 2003; Nieuwenhuijsen et al. 1999; Spaan et al. 2006). For these reasons, and for simplification of discussion by selecting a homogeneous population, studies of dairy farmers are the focus of this review.

Inverse associations with respiratory cancers have consistently been observed among dairy farmers (Laakkonen and Pukkala 2008; Mastrangelo et al. 2004, 2005; Pukkala and Notkola 1997; Reif et al. 1989; Stark et al. 1990; Wang et al. 2002) (Table 1). In a cohort of Italian dairy farmers, an inverse association with increased number of dairy cattle on the farm was demonstrated; a significant inverse trend (*p* = 0.001) was reported for farmers with more recent exposures (Mastrangelo et al. 2004, 2005). Lung and bronchus cancer risks were significantly lower among Finnish dairy farmers who continued farming at the time of follow-up (~ 20-year lag time) than for those that had quit farming, and risk of lung cancer was elevated for farmers who changed their production type to a crop or to beef cattle from the beginning of the study to follow-up, compared with those who continued as dairy farmers (Laakkonen and Pukkala 2008). An earlier follow-up from this same Finnish Farm Register base cohort also demonstrated a significant decrease in lung and bronchus cancer mortalities among dairy farmers and reported the risk was lowest among farmers with at least 10 dairy cows (Pukkala and Notkola 1997). Lung cancer mortality and incidence has also been shown to be significantly reduced in livestock farmers in the U.S. and Iceland, respectively (Gunnarsdóttir and Rafnsson 1991; Lange et al. 2003).

Only limited epidemiologic evidence is available from investigations of lung cancer risks in nontextile and nonfarming occupations that entail endotoxin exposure, yet the findings are generally consistent with an anticarcinogenic effect. Reduced lung cancer risks have been observed in U.S. automotive workers exposed to endotoxin from water-based metalworking fluids (Schroeder et al. 1997). The associations were primarily attributable to exposures within 10 years of death. Markedly reduced lung cancer incidence was also observed among pesticide applicators in the Agricultural Health Study cohort in the United States, which was attributed to a low prevalence of smoking habits (Alavanja et al. 2004; Blair et al. 2005). Pesticides were the principal focus of that study; endotoxin has not yet been investigated as a possible explanatory factor for the lung cancer deficit. A deficit in lung cancer risk was also observed in a study of more than a million Finnish men based on their self-reported longest held occupation in the 1970 national census, lagged by 20 years, with endotoxin exposure determined by an occupational exposure matrix (Laakkonen et al. 2008); a deficit was not observed in women. In contrast, a study of occupational exposures in Leningrad Province, Russia, reported a > 2-fold greater risk of lung cancer in subjects ever occupationally exposed to cotton dust (Baccarelli et al. 2006). Of note, the risk estimate was based on six cases, and the evaluation of cumulative exposure to cotton dust in males resulted in a protective effect.

Among the studies of endotoxin exposure and lung cancer, quantitative estimates of historical endotoxin exposures have been reconstructed for the Lithuanian (Kuzmickiene and Stukonis 2007) and Shanghai (Astrakianakis et al. 2006b, 2007) cohorts, and qualitative estimates of exposure have been estimated for Italian dairy farmers (Mastrangelo et al. 2005), to enable dose–response estimations of numerous site-specific cancers. All cohorts demonstrated a significant inverse dose response trend when evaluating endotoxin exposure by dust exposure category, cumulative cotton dust exposure, and number of dairy cattle on the farm, respectively, and lung cancer.

#### Other cancers

The findings to date for endotoxin exposure and risks for malignancies other than lung cancer have been limited and inconsistent. Much of the risk information on industrial exposures has been derived from the Shanghai cohort study of female textile workers. The first publication of this cohort described the occupational cancer risk for all textile workers, with select cancer outcomes evaluated by textile sector (Wernli et al. 2003). A decreased risk of most cancers was reported, with a significant decrease for esophageal, stomach, rectal, cervical, ovarian, and bladder cancers. Subsequent publications of this cohort evaluated the association of cumulative quantitative endotoxin exposure, as well as duration of occupational exposure classified by a job exposure matrix, and individual cancer end points, including liver, esophagus, stomach, rectum, pancreas, breast, brain, ovary, nasopharynx, and thyroid (Chang et al. 2006; De Roos et al. 2005; Gold et al. 2006; Li et al. 2006a, 2006b; Ray et al. 2007; Wernli et al. 2006, 2008; Wong et al. 2006). Notable findings from these studies include a decreased risk for cancer of the esophagus [hazard ratio (HR) = 0.5; 95% confidence interval (CI), 0.2–1.1] and increased risk for cancer of the nasopharynx (HR = 2.5; 95% CI, 1.1–5.4) (Li et al. 2006b; Wernli et al. 2006).

Other cotton textile industry cohorts have been evaluated for the association of occupational endotoxin exposure and cancers other than the lung. Szeszenia-Dabrowska (1999) reported a decreased risk of digestive cancers for men and women working in spinning and weaving departments. When considering individual cancers in men, there was a suggested increased risk of colon and liver cancers in weavers and stomach cancer in spinners, although these individual assessments were based on small numbers. Individual cancers in women showed a suggested decrease risk of rectal/anal and liver cancers and a suggested increase in gallbladder and ovarian cancers. In a Lithuanian cohort of textile workers, female workers in the spinning and weaving departments demonstrated increased risks for most individual cancers evaluated, with significant findings for breast and cervical cancers (Kuzmickiene et al. 2004). Other studies of cotton textile factory cohorts that defined exposure as employment in the production facility reported a decrease in breast and digestive cancers (Henderson and Enterline 1973; Hodgson and Jones 1990) and an increase in bladder, pharyngeal, and digestive cancers (Henderson and Enterline 1973; Mastrangelo et al. 2008). In a meta-analysis of 15 studies of cotton workers published during or before 1990, a nonsignificant increased risk of bladder cancer and decreased risk of digestive cancer were reported (Mastrangelo et al. 2002).

Among Finnish dairy farmers that continued farming at the time of follow-up (~ 20-year lag time), the risks of colon, liver, breast, bladder, and skin cancers were significantly decreased, and risk of lip cancer was significantly increased (Laakkonen and Pukkala 2008). Mastrangelo (2005) reported a decreased risk of mortality associated with most cancers evaluated in a cohort of Italian dairy farmers, with a significant decrease in esophageal, pancreatic, and bladder cancers. In a cohort of predominantly dairy farmers, female and male, in New York State, a decrease in risk was reported for most cancers, with significant decreases in risk for colon/rectum and ovarian cancers in females and cancers of the oral cavity, large intestine, and bladder in males (Stark et al. 1990; Wang et al. 2002).

### Physiologic response to endotoxin exposure and cancer risk

**Figure 1:**
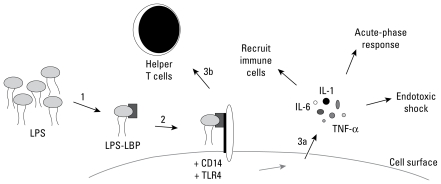
Mechanism of host response to LPS. Once internalized, LPS is bound by LBP (1) and transferred to CD14 (2); this new complex activates TLR4, followed by initiation of the innate (3a) and adaptive (3b) immune responses.

Various mechanistic arguments have been advanced regarding endotoxin and carcinogenesis, focusing largely on complex interactions between the innate and adaptive immune systems (Schmidt 2006; Tzianabos and Wetzler 2004). Once internalized, LPS is bound by LPS-binding protein (LBP) and then transferred to CD14 protein (Figure 1). The CD14–LPS complex binds to and activates the Toll-like receptors (TLRs), which are cell membrane signaling proteins located on cell surfaces of macrophages and other cells. TLR4 is the predominant receptor for endotoxin and is required for endotoxin recognition (Poltorak et al. 1998). Upon recognition of LPS, the innate inflammatory response is initiated and proinflammatory cytokines are released, including TNF-α, interleukin (IL)-1, and IL-6, which recruit immune cells to the site of exposure and induce the acute-phase response (Reisser et al. 2002; Tzianabos and Wetzler 2004). This host response is important for an effective immune system; however, overproduction of proinflammatory factors can cause endotoxic shock. In addition, TLR activation induces the expression of CD80 and CD86 on the surface of antigen-presenting cells that interact with the adaptive immune system to activate naive T-lymphocyte cells (T cells) (Heine et al. 2001; Hodgson 2006; Werling and Jungi 2003). The maturation of helper T cells (T_H_) results in cell-mediated (T_H_1) and humoral (T_H_2) subpopulations. The cytokines released by each of these cells have unique profiles and suppress the proliferation of the other subpopulation (Tzianabos and Wetzler 2004). The immune reaction to LPS primarily activates T_H_1 cells, which maximize the killing efficiency of macrophages and induce up-regulation of proinflammatory mediators (Heine et al. 2001; Lapa e Silva et al. 2000; Werling and Jungi 2003). Notably, antitumor activity has been related to the cytokine profile associated with a T_H_1 response, whereas the T_H_2 profile has been shown to be ineffective in eradicating tumors (Hong et al. 2008; Maraveyas et al. 1999).

#### Lung cancer

It has been postulated that bacterial endotoxin, through immunologic mechanisms, can be protective against lung cancer. Insofar as the route of endotoxin exposure is predominantly inhalation, the lung is one of the initial sites of immune stimulation (Liebers et al. 2006). Additionally, Klein et al. (1994) showed in a rat model that 5 min after injection of *Escherichia coli*, the 20% of bacteria not taken up by the liver were found in the lungs, spleen, and blood. The T_H_1 response favored by LPS-activated immune cells may be a conjectured benefit to this initial site of exposure in that the T_H_1 immune response tends to be more localized than the T_H_2 response (Tzianabos and Wetzler 2004). Moreover, the lung has been shown to produce or up-regulate the production of cofactors involved in the host response, including LBP, CD14, and TLR4, after LPS exposure (Fearns et al. 1995; Matsumura et al. 2000; Su et al. 1994). It is generally accepted that LBP is produced in the liver, but it has been shown that significant levels of LBP could be produced elsewhere in the body under induced conditions, such as an inflammatory response (Su et al. 1994). In the presence of LBP, approximately 15-fold less LPS have been reported to be required to trigger an inflammatory response, as measured using TNF-α (Martin et al. 1992; Schumann et al. 1990). There is also consistent experimental evidence for an increase in TNF in the bronchoalveolar lavage (BAL) fluid in guinea pigs after cotton dust exposure (Ryan and Karol 1991), and an increase in TNF in the BAL fluid of humans after endotoxin exposure (Jagielo et al. 1996; O’Grady et al. 2001; Wang et al. 1997). Likewise, Michel et al. (1997) reported a dose-dependent increase in TNF in the sputum of LPS-exposed subjects.

#### Other cancers

Other cancer end points have been studied, including cancers of the liver, esophagus, stomach, rectum, pancreas, breast, brain, ovary, thyroid, and nasopharynx, but not as extensively as the lung, and the findings have been inconsistent (Chang et al. 2006; De Roos et al. 2005; Gold et al. 2006; Henderson and Enterline 1973; Hodgson and Jones 1990; Kuzmickiene et al. 2004; Laakkonen et al. 2008; Li et al. 2006a; Mastrangelo et al. 2008; Merchant and Ortmeyer 1981; Ray et al. 2007; Stark et al. 1990; Szeszenia-Dabrowska et al. 1999; Wang et al. 2002; Wernli et al. 2003, 2006, 2008; Wong et al. 2006). Nonetheless, subsequent effects in other organ systems are plausible because cells with TLR4 receptors are widely disseminated, and elevation of systemic inflammatory mediators, including TNF-α, IL-1, IL-6, and IL-8, has been shown after inhalation of LPS or media contaminated with endotoxin (Hodgson 2006; Larsson et al. 1994; Mackensen et al. 1992; Mattsby and Rylander 1978; Michel et al. 1997; Palmberg et al. 2002; Reisser et al. 2002). Additionally, a dose-related systemic response to inhaled LPS in human subjects after bronchial challenges with pure LPS has been demonstrated (Michel et al. 1997).

## Discussion: Future Research Needs

The individual immune response to endotoxin is a complicated result of dose, timing, potential additive or synergistic effects, and genetically determined responsiveness (Liebers et al. 2008). The health effects, including cancer outcomes associated with exposure, remain paradoxical.

### Underlying biological mechanisms need to be elucidated

Insofar as endotoxin provokes an inflammatory response (Gordon 1992; Larsson et al. 1994; Mattsby and Rylander 1978; Michel et al. 1997), it might reasonably be anticipated that inflammation would enhance, rather than prevent, carcinogenesis (Bohnhorst et al. 2006; Puntoni et al. 2008; Schottenfeld and Beebe-Dimmer 2006). A sizable proportion of cancer deaths has been postulated to be attributable to infectious agents in which inflammation, mediated by recruitment of cytokines and growth factors to infected sites, may influence susceptibility to carcinogenesis through DNA damage and the simultaneous promotion of tissue destruction and repair (Schottenfeld and Beebe-Dimmer 2006). The roles of *H. pylori* (which generates endotoxin) in the etiology of adenocarcinoma of the stomach, human papillomavirus in the etiology of anogenital carcinoma, and hepatitis B or C virus in hepatocellular carcinoma are cases in point (Britton et al. 2005; Schottenfeld and Beebe-Dimmer 2006). Additionally, over-stimulation of inflammatory responses can lead to severe clinical symptoms, often termed sepsis, which can lead to progressive organ failure and death (Bosshart and Heinzelmann 2007). However, in lesser doses, which may relate best to chronic low-dose occupational and environmental endotoxin exposure, the proinflammatory mediators have been shown to inhibit tumor growth and retard tumor progression (Carswell et al. 1975; Dranoff 2004; Lin and Karin 2007; Manda et al. 1987).

Exposure to LPS has been demonstrated to induce pathologic hyperactivity (Suter and Kirsanow 1961), but a mechanism of protection from this lethal reactivity, termed endotoxin tolerance, has been speculated. Endotoxin tolerance is the unresolved phenomenon defined as an altered capacity to respond to LPS activation immediately after a first exposure; that is, when exposed to continual small doses of LPS, the same TNF response of the initial exposure does not necessarily occur with subsequent exposure (Cross 2002; Engelhardt et al. 1991; Gioannini et al. 2003; Michel et al. 1997; Otto et al. 1996; Palmberg et al. 2002). This tolerance has been shown to vary by dose as well as by length of time between treatments, and is theorized to allow the host more time to rid the pathogen (Cross 2002; Engelhardt et al. 1991). Because this tolerance has been related to allowing a body system to endure continuous small doses without adverse symptoms, a better understanding of this mechanism may bring clarity to the relationships between endotoxin sensitivity (including acute toxic effects) and sepsis, and, possibly, between carcinogenesis and protection against cancer (Cross 2002).

### Epidemiologic corroboration

Experimental evidence from both animal models and therapeutic trials regarding the effects of endotoxin on carcinogenic processes has not been consistent (Chen et al. 2007; Mumm and Oft 2008), which indicates the importance of epidemiologic observations for guiding mechanistic and clinical research. Difficulties in studying endotoxin epidemiologically include the very large degree of exposure variability over time and among study subjects, and uncertainties in the measurement, or proxy measure, of exposure (Spaan et al. 2008). The general pattern of endotoxin exposure and cancer that emerges from existing epidemiologic research is one suggestive of an anticarcinogenic effect of endotoxin exposure that occurs in the lung and, perhaps, other organs. This consistency of findings has been maintained when using job history as a proxy of exposure (Henderson and Enterline 1973; Hodgson and Jones 1990; Kuzmickiene and Stukonis 2007; Laakkonen and Pukkala 2008; Lange et al. 2003; Mastrangelo et al. 2008; Merchant and Ortmeyer 1981; Stark et al. 1990; Wang et al. 2002; Wernli et al. 2003), incorporating a cumulative endotoxin exposure matrix variable (Astrakianakis et al. 2007; Kuzmickiene and Stukonis 2007; Schroeder et al. 1997), and using number of dairy cattle on the farm (Mastrangelo et al. 2005). Nonetheless, with a few exceptions, most epidemiologic studies of endotoxin and cancer have not incorporated quantitative estimates of endotoxin exposure, which would strengthen causal arguments.

Although not unique to epidemiologic studies of endotoxin and cancer, absence of data on potentially confounding factors has been a limitation of most studies to date. Smoking status was incorporated in select analyses of endotoxin exposure and cancer and was shown to not account for the whole reduction in lung cancer risk, although the effect was exaggerated in those with low smoking habits (Astrakianakis et al. 2007; Blair et al. 2005; Hodgson and Jones 1990; Lange et al. 2003; Mastrangelo et al. 2004, 2005). Specifically, in the study of lung cancer among Shanghai textile workers, the inverse dose–response relation was not confounded by smoking, and importantly, the apparent protective effect was seen among both smokers and nonsmokers (Astrakianakis et al. 2007). The very low prevalence of smoking in this cohort of Chinese women workers precludes generalizability of these observations (Wernli et al. 2003), thus underscoring the importance of obtaining pertinent data on smoking and other cancer risk factors in future research.

## Concluding Remarks

Exposure to endotoxin is ubiquitous in the environment at levels that have been shown to have physiologic effects and, in some instances, demonstrable health consequences. There is very consistent epidemiologic evidence that endotoxin is dose-related to risk reductions for lung cancer, and provocative evidence that risks for other cancers may be similarly reduced. Animal experimental research and limited therapeutic trial data are generally supportive of an anticarcinogenesis effect, and plausible biological mechanisms have been described. The public health implications of findings to date could be substantial. Nevertheless, a more extensive assessment of the role of endotoxin in the etiology of cancers of the lung and other organs is needed. Future epidemiologic and toxicologic research to elucidate more precisely dose–response relations and underlying mechanisms will need to be conducted before endotoxin, an agent with established noncancer toxic health effects, could be considered for widespread chemoprevention uses (Boffetta 2007).

## Figures and Tables

**Table 1:** Lung cancer outcomes associated with occupational exposure to endotoxin.

			Outcomes			
			Overall		Highest exposure	
Location	Study	Sex	No. of cases	RR (95% CI)	No. of cases	RR (95% CI)
Cotton textile workers						
China	Astrakianakis et al. 2007^a^	F	—	—	74	0.70 (0.52–0.95)
China	Levin et al. 1987	M, F	169	0.7 (0.6–0.9)	48	0.8 (0.5–1.3)
China	Wernli et al. 2003^a^	F	641	0.80 (0.74–0.86)	236	0.72 (0.63–0.82)
Italy	Mastrangelo et al. 2008	M, F	36	1.03 (0.72–1.43)	10	0.93 (0.45–1.72)
Lithuania	Kuzmickiene and Stukonis 2007	M	70	0.94 (0.73–1.19)	2	0.24 (0.03–0.86)
		F	15	1.36 (0.76–2.25)	1	0.55 (0.01–3.08)
Poland	Szeszenia-Dabrowska et al. 1999	M	85	0.89 (0.71–1.10)	22	0.79 (0.50–1.20)
		F	12	0.55 (0.28–0.96)	9	0.82 (0.37–1.56)
UK	Hodgson and Jones 1990	M, F	42	0.76 (0.54–1.02)	—	—
USA	Henderson and Enterline 1973	M	20	0.55 (—)	—	—
USA	Merchant and Ortmeyer 1981	M	18	0.74 (—)	3	0.52 (—)

Dairy farmers						
Finland	Laakkonen and Pukkala 2008^b^	M, F	94	0.51 (0.42–0.62)	—	—
Finland	Pukkala and Notkola 1997^b^	M	185	0.5 (0.4–0.5)	—	—
		F	14	0.5 (0.3–0.8)	—	—
Italy	Mastrangelo et al. 2005	M	75	0.64 (0.51–0.81)	7	0.47 (0.19–0.96)
NZ	Reif et al. 1989	M	—	0.66 (0.48–0.92)	—	—
USA	Stark et al. 1990^c^	M	103	0.52 (—)	—	—
USA	Wang et al. 2002^c^	F	21	0.33 (0.20–0.51)	—	—

Abbreviations: —, data not available; F, female; M, male.

a Same cohort with different characterization of exposure.

b Same base cohort with different years of follow-up.

c Cohort of farm residents; > 50% were dairy farmers.

## References

[b1-ehp-117-1344] Alavanja MC, Dosemeci M, Samanic C, Lubin J, Lynch CF, Knott C (2004). Pesticides and lung cancer risk in the agricultural health study cohort. Am J Epidemiol.

[b2-ehp-117-1344] Andreani V, Gatti G, Simonella L, Rivero V, Maccioni M (2007). Activation of Toll-like receptor 4 on tumor cells in vitro inhibits subsequent tumor growth in vivo. Cancer Res.

[b3-ehp-117-1344] Astrakianakis G, Seixas N, Camp J, Smith TJ, Bartlett K, Checkoway H (2006a). Cotton dust and endotoxin levels in three Shanghai textile factories: a comparison of samplers. J Occup Environ Hygiene.

[b4-ehp-117-1344] Astrakianakis G, Seixas NS, Camp JE, Christiani DC, Feng Z, Thomas DB (2006b). Modeling, estimation and validation of cotton dust and endotoxin exposures in Chinese textile operations. Ann Occup Hygiene.

[b5-ehp-117-1344] Astrakianakis G, Seixas NS, Ray R, Camp JE, Gao DL, Feng Z (2007). Lung cancer risk among female textile workers exposed to endotoxin. J Natl Cancer Inst.

[b6-ehp-117-1344] Baccarelli A, Khmelnitskii O, Tretiakova M, Gorbanev S, Lomtev A, Klimkina I (2006). Risk of lung cancer from exposure to dusts and fibers in Leningrad Province, Russia. Am J Ind Med.

[b7-ehp-117-1344] Blair A, Sandler DP, Tarone R, Lubin J, Thomas K, Hoppin JA (2005). Mortality among participants in the agricultural health study. Ann Epidemiol.

[b8-ehp-117-1344] Boffetta P (2007). Endotoxins in lung cancer prevention. J Natl Cancer Inst.

[b9-ehp-117-1344] Bohnhorst J, Rasmussen T, Moen SH, Flottum M, Knudsen L, Borset M (2006). Toll-like receptors mediate proliferation and survival of multiple myeloma cells. Leukemia.

[b10-ehp-117-1344] Bosshart H, Heinzelmann M (2007). Targeting bacterial endotoxin: two sides of a coin. Ann N Y Acad Sci.

[b11-ehp-117-1344] Britton S, Papp-Szabo E, Simala-Grant J, Morrison L, Taylor DE, Monteiro MA (2005). A novel *Helicobacter pylori* cell-surface polysaccharide. Carbohydr Res.

[b12-ehp-117-1344] Campbell NA, Reece JB, Urry LA, Cain ML, Wasserman SA, Minorsky PV (2008). Biology.

[b13-ehp-117-1344] Carswell EA, Old LJ, Kassel RL, Green S, Fiore N, Williamson B (1975). An endotoxin-induced serum factor that causes necrosis of tumors. Proc Natl Acad Sci USA.

[b14-ehp-117-1344] CDC (1998). What You Need to Know about Occupational Exposure to Metalworking Fluids.

[b15-ehp-117-1344] CDC (Centers for Disease Control and Prevention) (2006). Health concerns associated with mold in water-damaged homes after hurricanes Katrina and Rita—New Orleans area, Louisiana, October 2005. MMWR Morb Mortal Wkly Rep.

[b16-ehp-117-1344] Chang CK, Astrakianakis G, Thomas DB, Seixas NS, Ray RM, Gao DL (2006). Occupational exposures and risks of liver cancer among Shanghai female textile workers—a case-cohort study. Int J Epidemiol.

[b17-ehp-117-1344] Chang CW, Chung H, Huang CF, Su HJ (2001). Exposure assessment to airborne endotoxin, dust, ammonia, hydrogen sulfide and carbon dioxide in open style swine houses. Ann Occup Hygiene.

[b18-ehp-117-1344] Chen R, Alvero AB, Silasi DA, Mor G (2007). Inflammation, cancer and chemoresistance: taking advantage of the Toll-like receptor signaling pathway. Am J Reprod Immunol.

[b19-ehp-117-1344] Chicoine MR, Won EK, Zahner MC (2001). Intratumoral injection of lipopolysaccharide causes regression of subcutaneously implanted mouse glioblastoma multiforme. Neurosurgery.

[b20-ehp-117-1344] Clark IA (2007). How TNF was recognized as a key mechanism of disease. Cytokine Growth Factor Rev.

[b21-ehp-117-1344] Coley WB (1894). Treatment of inoperable malignant tumors with the toxins of erysipelas and the Bacillus prodigiosus. Trans Am Surg Assn.

[b22-ehp-117-1344] Cross AS (2002). Endotoxin tolerance-current concepts in historical perspective. J Endotoxin Res.

[b23-ehp-117-1344] de Bono JS, Dalgleish AG, Carmichael J, Diffley J, Lofts FJ, Fyffe D (2000). Phase I study of ONO-4007, a synthetic analogue of the lipid A moiety of bacterial lipopolysaccharide. Clin Cancer Res.

[b24-ehp-117-1344] De Roos AJ, Ray RM, Gao DL, Wernli KJ, Fitzgibbons ED, Ziding F (2005). Colorectal cancer incidence among female textile workers in Shanghai, China: a case-cohort analysis of occupational exposures. Cancer Causes Control.

[b25-ehp-117-1344] Dranoff G (2004). Cytokines in cancer pathogenesis and cancer therapy. Nat Rev.

[b26-ehp-117-1344] Eduard W, Omenaas E, Bakke PS, Douwes J, Heederik D (2004). Atopic and non-atopic asthma in a farming and a general population. Am J Indust Med.

[b27-ehp-117-1344] Engelhardt R, Mackensen A, Galanos C (1991). Phase I trial of intravenously administered endotoxin (*Salmonella abortus equi*) in cancer patients. Cancer Res.

[b28-ehp-117-1344] Fearns C, Kravchenko VV, Ulevitch RJ, Loskutoff DJ (1995). Murine CD14 gene expression in vivo: extramyeloid synthesis and regulation by lipopolysaccharide. J Exp Med.

[b29-ehp-117-1344] Gehring U, Bischof W, Schlenvoigt G, Richter K, Fahlbusch B, Wichmann HE (2004). Exposure to house dust endotoxin and allergic sensitization in adults. Allergy.

[b30-ehp-117-1344] Ghezzi P, Cerami A (2005). Tumor necrosis factor as a pharmacological target. Mol Biotechnol.

[b31-ehp-117-1344] Gioannini TL, Teghanemt A, Zarember KA, Weiss JP (2003). Regulation of interactions of endotoxin with host cells. J Endotoxin Res.

[b32-ehp-117-1344] Gold LS, De Roos AJ, Ray RM, Wernli K, Fitzgibbons ED, Gao DL (2006). Brain tumors and occupational exposures in a cohort of female textile workers in Shanghai, China. Scand J Work Environ Health.

[b33-ehp-117-1344] Gordon T (1992). Dose-dependent pulmonary effects of inhaled endotoxin in guinea pigs. Environ Res.

[b34-ehp-117-1344] Goto S, Sakai S, Kera J, Suma Y, Soma GI, Takeuchi S (1996). Intradermal administration of lipopolysaccharide in treatment of human cancer. Cancer Immunol Immunother.

[b35-ehp-117-1344] Gunnarsdóttir H, Rafnsson V (1991). Cancer incidence among Icelandic farmers 1977–1987. Scand J Soc Med.

[b36-ehp-117-1344] Heine H, Rietschel ET, Ulmer AJ (2001). The biology of endotoxin. Mol Biotechnol.

[b37-ehp-117-1344] Henderson V, Enterline PE (1973). An unusual mortality experience in cotton textile workers. J Occup Med.

[b38-ehp-117-1344] Hodgson JC (2006). Endotoxin and mammalian host responses during experimental disease. J Comp Pathol.

[b39-ehp-117-1344] Hodgson JT, Jones RD (1990). Mortality of workers in the British cotton industry in 1968–1984. Scand J Work Environ Health.

[b40-ehp-117-1344] Hong S, Qian J, Yang J, Li H, Kwak LW, Yi Q (2008). Roles of idio-type-specific T cells in myeloma cell growth and survival: Th1 and CTL cells are tumoricidal while Th2 cells promote tumor growth. Cancer Res.

[b41-ehp-117-1344] Jagielo PJ, Thorne PS, Watt JL, Frees KL, Quinn TJ, Schwartz DA (1996). Grain dust and endotoxin inhalation challenges produce similar inflammatory responses in normal subjects. Chest.

[b42-ehp-117-1344] Klein A, Zhadkewich M, Margolick J, Winkelstein J, Bulkley G (1994). Quantitative discrimination of hepatic reticulo-endothelial clearance and phagocytic killing. J Leukocyte Biol.

[b43-ehp-117-1344] Koskela RS, Klockars M, Järvinen E (1990). Mortality and disability among cotton mill workers. Br J Ind Med.

[b44-ehp-117-1344] Kuramitsu Y, Nishibe M, Ohiro Y, Matsushita K, Yuan L, Obara M (1997). A new synthetic lipid A analog, ONO-4007, stimulates the production of tumor necrosis factor-alpha in tumor tissues, resulting in the rejection of transplanted rat hepatoma cells. Anticancer Drugs.

[b45-ehp-117-1344] Kuzmickiene I, Didziapetris R, Stukonis M (2004). Cancer incidence in the workers cohort of textile manufacturing factory in Alytus, Lithuania. J Occup Environ Med.

[b46-ehp-117-1344] Kuzmickiene I, Stukonis M (2007). Lung cancer risk among textile workers in Lithuania. J Occup Med Toxicol.

[b47-ehp-117-1344] Laakkonen A, Pukkala E (2008). Cancer incidence among Finnish farmers, 1995–2005. Scand J Work Environ Health.

[b48-ehp-117-1344] Laakkonen A, Verkasalo PK, Nevalainen A, Kauppinen T, Kyyronen P, Pukkala EI (2008). Moulds, bacteria and cancer among Finns: an occupational cohort study. Occup Environ Med.

[b49-ehp-117-1344] Lange JH (1992). An experimental study of anti-cancer properties of aerosolized endotoxin: application to human epidemiological studies. J Occup Med Toxicol.

[b50-ehp-117-1344] Lange JH, Mastrangelo G, Fedeli U, Fadda E, Rylander R, Lee E (2003). Endotoxin exposure and lung cancer mortality by type of farming: is there a hidden dose-response relationship?. Ann Agric Environ Med.

[b51-ehp-117-1344] Lapa e Silva JR, Possebon da Silva MD, Lefort J, Vargaftig BB (2000). Endotoxins, asthma, and allergic immune responses. Toxicology.

[b52-ehp-117-1344] Larsson KA, Eklund AG, Hansson LO, Isaksson BM, Malmberg PO (1994). Swine dust causes intense airways inflammation in healthy subjects. Am J Respir Crit Care Med.

[b53-ehp-117-1344] Lee E, Burnett CA, Lalich N, Cameron LL, Sestito JP (2002). Proportionate mortality of crop and livestock farmers in the United States, 1984–1993. Am J Ind Med.

[b54-ehp-117-1344] Levin LI, Gao YT, Blot WJ, Zheng W, Fraumeni JF (1987). Decreased risk of lung cancer in the cotton textile industry of Shanghai. Cancer Res.

[b55-ehp-117-1344] Li W, Ray RM, Gao DL, Fitzgibbons ED, Seixas NS, Camp JE (2006a). Occupational risk factors for pancreatic cancer among female textile workers in Shanghai, China. Occup Environ Med.

[b56-ehp-117-1344] Li W, Ray RM, Gao DL, Fitzgibbons ED, Seixas NS, Camp JE (2006b). Occupational risk factors for nasopharyngeal cancer among female textile workers in Shanghai, China. Occup Environ Med.

[b57-ehp-117-1344] Liebers V, Bruning T, Raulf-Heimsoth M (2006). Occupational endotoxin-exposure and possible health effects on humans. Am J Indust Med.

[b58-ehp-117-1344] Liebers V, Raulf-Heimsoth M, Bruning T (2008). Health effects due to endotoxin inhalation. Arch Toxicol.

[b59-ehp-117-1344] Lin WW, Karin M (2007). A cytokine-mediated link between innate immunity, inflammation, and cancer. J Clin Invest.

[b60-ehp-117-1344] Liu AH (2002). Endotoxin exposure in allergy and asthma: reconciling a paradox. J Allergy Clin Immunol.

[b61-ehp-117-1344] Mackensen A, Galanos C, Wehr U, Engelhardt R (1992). Endotoxin tolerance: regulation of cytokine production and cellular changes in response to endotoxin application in cancer patients. EurCytokine Netw.

[b62-ehp-117-1344] Manda T, Shimomura K, Mukumoto S, Kobayashi K, Mizota T, Hirai O (1987). Recombinant human tumor necrosis factor-alpha: evidence of an indirect mode of antitumor activity. Cancer Res.

[b63-ehp-117-1344] Mandryk J, Alwis KU, Hocking AD (1999). Work-related symptoms and dose-response relationships for personal exposures and pulmonary function among woodworkers. Am J Indust Med.

[b64-ehp-117-1344] Maraveyas A, Baban B, Kennard D, Rook GA, Westby M, Grange JM (1999). Possible improved survival of patients with stage IV AJCC melanoma receiving SRL 172 immunotherapy: correlation with induction of increased levels of intracellular interleukin-2 in peripheral blood lymphocytes. Ann Oncol.

[b65-ehp-117-1344] Martin TR, Mathison JC, Tobias PS, Leturcq DJ, Moriarty AM, Maunder RJ (1992). Lipopolysaccharide binding protein enhances the responsiveness of alveolar macrophages to bacterial lipopolysaccharide. Implications for cytokine production in normal and injured lungs. J Clin Invest.

[b66-ehp-117-1344] Mastrangelo G, Fadda E, Rylander R, Milan G, Fedeli U, Rossi di Schio M (2008). Lung and other cancer site mortality in a cohort of Italian cotton mill workers. Occup Environ Med.

[b67-ehp-117-1344] Mastrangelo G, Fedeli U, Fadda E, Milan G, Lange JH (2002). Epidemiologic evidence of cancer risk in textile industry workers: a review and update. Toxicol Ind Health.

[b68-ehp-117-1344] Mastrangelo G, Grange JM, Fadda E, Fedeli U, Buja A, Lange JH (2005). Lung cancer risk: effect of dairy farming and the consequence of removing that occupational exposure. Am J Epidemiol.

[b69-ehp-117-1344] Mastrangelo G, Marzia V, Marcer G (1996). Reduced lung cancer mortality in dairy farmers: is endotoxin exposure the key factor?. Am J Ind Med.

[b70-ehp-117-1344] Mastrangelo G, Marzia V, Milan G, Fadda E, Fedeli U, Lange JH (2004). An exposure-dependent reduction of lung cancer risk in dairy farmers: a nested case-referent study. Indoor Built Environ.

[b71-ehp-117-1344] Matsumura T, Ito A, Takii T, Hayashi H, Onozaki K (2000). Endotoxin and cytokine regulation of Toll-like receptor (TLR) 2 and TLR4 gene expression in murine liver and hepatocytes. J Interferon Cytokine Res.

[b72-ehp-117-1344] Mattsby I, Rylander R (1978). Clinical and immunological findings in workers exposed to sewage dust. J Occup Med.

[b73-ehp-117-1344] McCarthy EF (2006). The toxins of William B. Coley and the treatment of bone and soft-tissue sarcomas. Iowa Orthop J.

[b74-ehp-117-1344] Merchant JA, Ortmeyer C (1981). Mortality of employees of two cotton mills in North Carolina. Chest.

[b75-ehp-117-1344] Michel O, Kips J, Duchateau J, Vertongen F, Robert L, Collet H (1996). Severity of asthma is related to endotoxin in house dust. Am J Respir Crit Care Med.

[b76-ehp-117-1344] Michel O, Nagy AM, Schroeven M, Duchateau J, Neve J, Fondu P (1997). Dose-response relationship to inhaled endotoxin in normal subjects. Am J Respir Crit Care Med.

[b77-ehp-117-1344] Morita S, Yamamoto M, Kamigaki T, Saitoh Y (1996). Synthetic lipid A produces antitumor effect in a hamster pancreatic carcinoma model through production of tumor necrosis factor from activated macrophages. Kobe J Med Sci.

[b78-ehp-117-1344] Mueller H (1998). Tumor necrosis factor as an antineoplastic agent: pitfalls and promises. Cell Mol Life Sci.

[b79-ehp-117-1344] Mumm JB, Oft M (2008). Cytokine-based transformation of immune surveillance into tumor-promoting inflammation. Oncogene.

[b80-ehp-117-1344] National Library of Medicine (2006). Medline.

[b81-ehp-117-1344] Nieuwenhuijsen MJ, Noderer KS, Schenker MB, Vallyathan V, Olenchock S (1999). Personal exposure to dust, endotoxin and crystalline silica in California agriculture. Ann Occup Hygiene.

[b82-ehp-117-1344] O’Grady NP, Preas HL, Pugin J, Fiuza C, Tropea M, Reda D (2001). Local inflammatory responses following bronchial endotoxin instillation in humans. Am J Respir Crit Care Med.

[b83-ehp-117-1344] Otto F, Schmid P, Mackensen A, Wehr U, Seiz A, Braun M (1996). Phase II trial of intravenous endotoxin in patients with colorectal and non-small cell lung cancer. Eur J Cancer.

[b84-ehp-117-1344] Palmberg L, Larssson BM, Malmberg P, Larsson K (2002). Airway responses of healthy farmers and nonfarmers to exposure in a swine confinement building. Scand J Work Environ Health.

[b85-ehp-117-1344] Park JH, Cox-Ganser J, Rao C, Kreiss K (2006). Fungal and endotoxin measurements in dust associated with respiratory symptoms in a water-damaged office building. Indoor Air.

[b86-ehp-117-1344] Poltorak A, He X, Smirnova I, Liu MY, Van Huffel C, Du X (1998). Defective LPS signaling in C3H/HeJ and C57BL/10ScCr mice: mutations in Tlr4 gene. Science.

[b87-ehp-117-1344] Portengen L, Preller L, Tielen M, Doekes G, Heederik D (2005). Endotoxin exposure and atopic sensitization in adult pig farmers. J Allergy Clin Immunol.

[b88-ehp-117-1344] Pukkala E, Notkola V (1997). Cancer incidence among Finnish farmers, 1979–93. Cancer Causes Control.

[b89-ehp-117-1344] Puntoni M, Marra D, Zanardi S, Decensi A (2008). Inflammation and cancer prevention. Ann Oncol.

[b90-ehp-117-1344] Rapiti E, Sperati A, Fano V, Dell’Orco V, Forastiere F (1997). Mortality among workers at municipal waste incinerators in Rome: a retrospective cohort study. Am J Ind Med.

[b91-ehp-117-1344] Ray RM, Gao DL, Li W, Wernli KJ, Astrakianakis G, Seixas NS (2007). Occupational exposures and breast cancer among women textile workers in Shanghai. Epidemiology.

[b92-ehp-117-1344] Reif J, Pearce N, Fraser J (1989). Cancer risks in New Zealand farmers. Int J Epidemiol.

[b93-ehp-117-1344] Reisser D, Pance A, Jeannin JF (2002). Mechanisms of the anti-tumoral effect of lipid A. Bioessays.

[b94-ehp-117-1344] Remes ST, Iivanainen K, Koskela H, Pekkanen J (2003). Which factors explain the lower prevalence of atopy amongst farmers’ children?. Clin Exp Allergy.

[b95-ehp-117-1344] Ryan LK, Karol MH (1991). Release of tumor necrosis factor in guinea pigs upon acute inhalation of cotton dust. Am J Resp Cell Mol Biol.

[b96-ehp-117-1344] Rylander R (2002). Endotoxin in the environment—exposure and effects. J Endotoxin Res.

[b97-ehp-117-1344] Rylander R (2006). Endotoxin and occupational airway disease. Curr Opin Allergy Clin Immunol.

[b98-ehp-117-1344] Rylander R, Sorenson S, Gotoo H, Yusao K, Tanaka S, Bieva CJ, Courtois Y, Govaerts M (1989). The importance of endotoxin and glucan for symptoms in sick buildings. Present and Future of Indoor Air Quality.

[b99-ehp-117-1344] Schmidt C (2006). Immune system’s Toll-like receptors have good opportunity for cancer treatment. J Natl Cancer Inst.

[b100-ehp-117-1344] Schottenfeld D, Beebe-Dimmer J (2006). Chronic inflammation: a common and important factor in the pathogenesis of neoplasia. CA Cancer J Clin.

[b101-ehp-117-1344] Schroeder JC, Tolbert PE, Eisen EA, Monson RR, Hallock MF, Smith TJ (1997). Mortality studies of machining fluid exposure in the automobile industry. IV: A case-control study of lung cancer. Am J Indust Med.

[b102-ehp-117-1344] Schumann RR, Leong SR, Flaggs GW, Gray PW, Wright SD, Mathison JC (1990). Structure and function of lipopolysaccharide binding protein. Science.

[b103-ehp-117-1344] Schwartz DA, Donham KJ, Olenchock SA, Popendorf WJ, Van Fossen DS, Burmeister LF (1995). Determinants of longitudinal changes in spirometric function among swine confinement operators and farmers. Am J Respir Crit Care Med.

[b104-ehp-117-1344] Shear MJ, Perrault A (1944). Reactions of mice with primary subcutaneous tumors to the injection of a hemorrhage-producing bacterial polysaccharide. J Natl Cancer Inst.

[b105-ehp-117-1344] Shear MJ, Turner FC (1943). Chemical treatment of tumours; isolation of hemorrhagic-producing fraction from *Serratia marcescens* (*Bacillus prodigious*) culture filtrate. J Natl Cancer Inst.

[b106-ehp-117-1344] Smid T, Heederik D, Houba R, Quanjer PH (1992). Dust- and endotoxin-related respiratory effects in the animal feed industry. Am Rev Respir Dis.

[b107-ehp-117-1344] Spaan S, Schinkel J, Wouters IM, Preller L, Tielemans E, Nij ET (2008). Variability in endotoxin exposure levels and consequences for exposure assessment. Ann Occup Hyg.

[b108-ehp-117-1344] Spaan S, Wouters IM, Oosting I, Doekes G, Heederik D (2006). Exposure to inhalable dust and endotoxins in agricultural industries. J Environ Monit.

[b109-ehp-117-1344] Spriggs DR, Sherman ML, Michie H, Arthur KA, Imamura K, Wilmore D (1988). Recombinant human tumor necrosis factor administered as a 24-hour intravenous infusion. A phase 1 and pharmacologic study. J Natl Cancer Inst.

[b110-ehp-117-1344] Stark AD, Chang HG, Fitzgerald EF, Riccardi K, Stone RR (1990). A retrospective cohort study of cancer incidence among New York State Farm Bureau members. Arch Environ Health.

[b111-ehp-117-1344] Su GL, Freeswick PD, Geller DA, Wang Q, Shapiro RA, Wan YH (1994). Molecular cloning, characterization, and tissue distribution of rat lipopolysaccharide binding protein. Evidence for extrahepatic expression. J Immunol.

[b112-ehp-117-1344] Suter E, Kirsanow EM (1961). Hyperreactivity to endotoxin in mice infected with Mycobacterium. Induction and elicitation of the reaction. Immunology.

[b113-ehp-117-1344] Szeszenia-Dabrowska N, Wilczynska U, Strzelecka A, Sobala W (1999). Mortality in the cotton industry workers: results of a cohort study. Int J Occup Med Environ Health.

[b114-ehp-117-1344] Tzianabos AO, Wetzler LM, Pier GB, Lyczak JB, Wetzler LM (2004). Cellular communication. Immunology, Infection, and Immunity.

[b115-ehp-117-1344] von Mutius E, Braun-Fahrlander C, Schierl R, Riedler J, Ehlermann S, Maisch S (2000). Exposure to endotoxin or other bacterial components might protect against the development of atopy. Clin Exp Allergy.

[b116-ehp-117-1344] Wang XR, Zhang HX, Sun BX, Dai HL, Hang JQ, Eisen EA (2005). A 20-year follow-up study on chronic respiratory effects of exposure to cotton dust. Eur Respir J.

[b117-ehp-117-1344] Wang Y, Lewis-Michl EL, Hwang SA, Fitzgerald EF, Stark AD (2002). Cancer incidence among a cohort of female farm residents in New York State. Arch Environ Health.

[b118-ehp-117-1344] Wang Z, Larsson K, Palmberg L, Malmberg P, Larsson P, Larsson L (1997). Inhalation of swine dust induces cytokine release in the upper and lower airways. Eur Respir J.

[b119-ehp-117-1344] Werling D, Jungi TW (2003). TOLL-like receptors linking innate and adaptive immune response. Vet Immunol Immunopathol.

[b120-ehp-117-1344] Wernli KJ, Fitzgibbons ED, Ray RM, Gao DL, Li W, Seixas NS (2006). Occupational risk factors for esophageal and stomach cancers among female textile workers in Shanghai, China. Am J Epidemiol.

[b121-ehp-117-1344] Wernli KJ, Ray RM, Gao DL, Fitzgibbons ED, Camp JE, Astrakianakis G (2008). Occupational exposures and ovarian cancer in textile workers. Epidemiology.

[b122-ehp-117-1344] Wernli KJ, Ray RM, Gao DL, Thomas DB, Checkoway H (2003). Cancer among women textile workers in Shanghai, China: overall incidence patterns, 1989–1998. Am J Indust Med.

[b123-ehp-117-1344] Won EK, Zahner MC, Grant EA, Gore P, Chicoine MR (2003). Analysis of the antitumoral mechanisms of lipopolysaccharide against glioblastoma multiforme. Anticancer Drugs.

[b124-ehp-117-1344] Wong EY, Ray R, Gao DL, Wernli KJ, Li W, Fitzgibbons ED (2006). Reproductive history, occupational exposures, and thyroid cancer risk among women textile workers in Shanghai, China. Int Arch Occup Environ Health.

[b125-ehp-117-1344] Wouters IM, Spaan S, Douwes J, Doekes G, Heederik D (2006). Overview of personal occupational exposure levels to inhalable dust, endotoxin, β(1→3)-glucan and fungal extracellular polysaccharides in the waste management chain. Ann Occup Hygiene.

